# Investigating the feasibility and acceptability of using Instagram to engage post-graduate students in a mass communication social media-based health intervention, #WeeStepsToHealth

**DOI:** 10.1186/s40814-022-01207-9

**Published:** 2022-12-12

**Authors:** Niamh O’Kane, Michelle C. McKinley, Aisling Gough, Ruth F. Hunter

**Affiliations:** Centre for Public Health, Queen’s University Belfast, Belfast, BT12 6BJ UK

**Keywords:** Social media, Public health, Physical activity, Nutrition, Instagram, Intervention, Feasibility

## Abstract

**Background:**

Instagram’s popularity among young adults continues to rise, and previous work has identified diffusion of unhealthy messages and misinformation throughout the platform. However, we know little about how to use Instagram to promote health messages. This study aims to assess the feasibility and acceptability of using Instagram to engage post-graduate students in a mass communication social media (SM)-based health intervention.

**Methods:**

A 4-week intervention targeting post-graduate students with physical activity (PA), nutrition, and general wellbeing messages was conducted via Instagram. Feasibility and acceptability were assessed using SM metrics (likes, comments, and shares), pre- and post-intervention online surveys (knowledge, attitude, and behavioural outcomes), and a focus group conducted with a sample of individuals in the target population (to assess intervention recall, feedback on message framing, and acceptability of Instagram)*.*

**Results:**

The two independent samples captured by online surveys (pre-intervention, *n* = 43, post-intervention, *n* = 41, representing 12.3% and 11.7% of Instagram followers, respectively) were predominantly female (88.4%, 80.5%) aged 18–34 (95.4%, 95.1%). Respondents in the second survey reported higher weekly PA levels (+ 13.7%) and more frequent nutritional behaviours including consumption of five or more fruits and vegetables (+ 23.3%) and looking at nutritional labels (+ 10.3%). However, respondents in the second survey also reported less frequent meal preparation (− 18.0%) and a small increase in fast food consumption (+ 2.8% consuming fast food 3–4 days a week). A total of 247 ‘likes’ were collected from 28 Instagram posts (mean 8.8 likes per post). Humorous posts achieved a moderately higher level of engagement than non-humorous posts (median 10 and 8 likes, respectively). Focus group participants liked the campaign content and trusted the information source.

**Conclusions:**

Findings indicate that Instagram could be a feasible and acceptable platform for engaging post-graduate students in a SM-based mass communication health intervention, and that humour may have the potential to encourage further engagement.

**Supplementary information:**

The online version contains supplementary material available at 10.1186/s40814-022-01207-9.

## Key messages regarding feasibility

• Social media (SM) interventions are relatively novel, and there is no clear guidance on conducting a SM-based intervention, particularly on visual SM platforms such as Instagram. It is thus far unclear as to whether Instagram is a feasible platform for engaging post-graduate students in an intervention designed to improve nutrition, physical activity, and overall wellbeing. Specific uncertainties include the following: (i) feasibility of using an Instagram intervention to incite behaviour change, knowledge, or attitudes among a student population; (ii) the collection of SM metrics (such as likes, comments) for assessing engagement with the intervention; and (iii) the use of humour to improve this engagement.

• The key feasibility findings are as follows: (i) The study found Instagram to be a feasible platform for nutrition, physical activity, and wellbeing intervention for students: the post-intervention survey showed moderately higher reported levels of physical activity and positive nutritional behaviours (such as consuming five fruit and veg a day and actively looking at nutritional labels); (ii) SM metrics were a feasible data collection method for measuring engagement with the intervention content, as explored further in post-intervention focus groups with students; and (iii) the content (including humorous content) was found to be acceptable for engaging students online.

• The study findings demonstrate the feasibility of using Instagram as a platform for disseminating a mass-communication social media intervention targeting post-graduate students with nutrition, physical activity, and general wellbeing information. This should be further explored in a controlled pilot study in a larger student population, following Medical Research Council (MRC) guidance for developing and evaluating complex interventions.

## Background

### Instagram as a social media platform for public health messages

Whilst the growth of some social media (SM) platforms has begun to plateau, the popularity of visual SM platforms, such as Instagram and Pinterest, continues to rise [1]. Instagram is particularly popular among young adults ranging from 16 to 34 years of age [1, 2], but there is an increasing body of research into content and advertising on Instagram, suggesting that Instagram may promote the diffusion of unhealthy behaviours [3–5]. Instagram is used by corporations to target audiences with unhealthy products, such as alcohol, to users as young as 13 years old [6]. Thus, there is a responsibility placed on the public health research community to explore the use of this platform for the diffusion of positive health messages and to combat the diffusion of unhealthy behaviours.

### Health behaviours of university students

Studies have shown significant increases in weight and body mass index (BMI) in students throughout the course of a degree, with an increase in stressors, changing eating habits, and moving away from home suggested as contributors to weight gain and changes in lifestyle behaviours [7–10]. If such trends continue post-university, this could have negative implications for long-term health [10]. A number of studies recommend targeting students’ physical activity (PA) and nutrition behaviours (i.e. weight-related behaviours) as a means of maintaining a healthy weight status, increasing physical self-esteem, and improving mental wellbeing [11, 12]. Additionally, previous SM-based health interventions suggest SM platforms to be potentially effective platforms for increasing knowledge in this population, specifically regarding risky sexual behaviour, and in the improvement of nutrition outcomes [13, 14]. Therefore, the intervention in the current study aimed to explore the feasibility and acceptability of Instagram for disseminating evidence-based, positive health messages regarding PA, nutrition, and general wellbeing issues relevant to this population.

### Message framing in public health interventions

Employing SM platforms to disseminate health interventions offers a level of dynamic and targeted messaging not always possible with traditional mass communication [15]. Instagram can deliver messages with different frames, such as positive framing, negative framing, and humour, meaning it can be used to target various audiences that may respond to different message frames [16, 17]. The implementation of different message frames in SM-based health interventions has been explored in previous studies, including the use of shock [18, 19] and humour [19, 20]. Humorous messages achieved a higher level of engagement, suggesting that humour on SM may promote engagement with health promotion content [21]. Thus, a secondary aim of this study was to explore if humorous posts encourage higher levels of SM engagement.

## Materials and methods

A mixed-methods approach was used to investigate the following: (1) feasibility of collecting data on population characteristics, knowledge, attitudes, and self-reported behaviour pre and post intervention, (2) feasibility of collecting data on engagement with intervention using SM metrics, and (3) acceptability of the campaign. The above provided indication of feasibility of data collection methods and acceptability of Instagram as a health promotion platform. Quantitative data (SM metrics and online survey) and qualitative data (online survey and in-person focus group) were collected.

### Intervention development

#### Theoretical underpinning

Intervention development was guided by the Medical Research Council (MRC) framework for developing and evaluating complex interventions [22]; relevant theory was identified to underpin the work, content was piloted among a sample of the population (post-graduate students), and subsequent modifications were made before implementation. Post-graduate students were chosen as the target population as the university has a specific school (and therefore SM channel) dedicated to post-graduate students, which is potentially less likely to be followed by non-students or other irrelevant audiences compared to the general university channel (of which obtaining the rights to disseminate content would have proven difficult in addition). The diffusion of innovations theory explains how new ideas and practices (or beliefs and behaviours) spread within and between communities, diffusing via interpersonal contact [23, 24]. Hence, the theory posits a model for intervention dissemination, whereby intervention content proliferates through existing social networks (in this case, the followers of Queen’s University Belfast’s ‘Graduate School’ Instagram page).

Intervention content was developed using behaviour change techniques (BCTs) to guide creation, with a specific focus on information about health consequences (i.e. providing information about consequences of performing behaviours, such as eating junk food), practical social support (i.e. advising on practical help for performance of behaviour, such as encouraging followers to take a study break with friends), and behaviour substitution (i.e. suggesting alternatives to current behaviours, such as walking a longer route to lunch). These are techniques suggested to be effective BCTs in previous studies among this population [14, 25, 26]. Topics (PA, nutrition, and general wellbeing) were chosen as previous studies have recommended targeting these areas in university students, in order to aid in maintaining a healthy weight status, increasing physical self-esteem, and improving mental wellbeing [11, 12]. Information provided in the SM posts was evidence based, and all claims were supported by inclusion of sources at the end of each post’s caption.

#### Message frame

In order to observe whether humour would encourage engagement with the posts, two different frames were delivered: (i) educational or informative posts delivering a visual health message and (ii) humorous educational posts, incorporating memes and popular culture. All content included the unique hashtag ‘#WeeStepsToHealth’, a name reflecting the focus of the Instagram posts (providing ‘small steps’ to improving health) but also incorporating a local Irish colloquialism (‘wee’). Please see supplementary material file 1 for examples of content.

#### Qualitative field testing

The MRC guidelines for complex interventions and previous work by the research team were used to guide message development [19, 22]. Preliminary content was field tested among the population in a workshop with a small sample (*n* = 5, four females, one male) of post-graduate students, and feedback was used to refine the content further. For example, participants were keen on ‘substitution’ posts for dietary choices, found ‘memes’ funnier than cartoons, and found nutrition-related posts more interesting than PA-related posts. Therefore, substitution-style posts were increased in number, cartoons in content were replaced with memes, and content focused more on nutrition than PA.

### Design and implementation

The intervention was delivered over a 4-week period using the Queen’s University Belfast (QUB) Graduate School’s Instagram account as an ‘information intermediary’ (i.e. an account not considered health professional but which still has an established credibility). The Graduate School’s content schedule was not affected by the intervention; thus, intervention content was posted intermittently between unrelated posts, such as advertisements for events. A number of posts were also cross-posted across the school’s Facebook, Twitter, or both, where The Graduate School deemed it appropriate.

### Pre- and post-intervention online surveys

Pre- and post-intervention surveys were hosted on SurveyMonkey (surveymonkey.co.uk) and advertised through The Graduate School’s Instagram and Twitter, capturing two independent samples. Please see supplementary material file 2 for survey. Whilst independent samples makes comparison pre- and post-intervention difficult, this approach was chosen because the aim was to assess the feasibility of using Instagram as an intervention vehicle. In a real-world instance of the use of SM recruitment for two surveys, there is no guarantee of capturing the same audience twice; therefore, it was deemed inappropriate to recruit the same sample of respondents to complete the two surveys, as to do so would have limited applications.

Surveys assessed population characteristics, knowledge and attitudes towards general health issues, PA, and nutrition behaviours, as well as SM usage. Validated measures were used to capture PA in the last week/month (in days) [27], and respondents were quizzed on knowledge of recommended PA guidelines. For example, respondents were asked to identify how many minutes of moderate to vigorous physical activity (MVPA) they believed was the recommended for adults. If respondents answered 150 min of MVPA, this was identified as correct, with over and below this figure scoring as overestimate and underestimate respectively. Nutrition-related questions asked respondents about calories, nutritional labels, fruits and vegetables, fast food consumption, and meal preparation. Respondents were asked about attitudes towards general health-related issues (sleep, smoking, and stress). Finally, SM habits were collected from respondents on platform use.

#### Sample size

The minimum response rate was set at 10% of Instagram followers, calculated by adapting the concept of ‘view rate’ from the Checklist for Reporting Results of Internet E-Surveys (CHERRIES) [28] (i.e. ratio unique site visitors/unique survey visitors) to the ratio of Instagram followers to unique survey visitors. The baseline number of followers for the page was 350; thus, the minimum acceptable response rate was 35 participants. Response rate was determined in regard to Instagram followers as it was the primary dissemination platform, and thus, number of followers of The Graduate School’s Facebook and Twitter account was not collected. Respondents to each survey were entered into a lottery for Amazon vouchers, with three winners selected at random.

### Focus group

One focus group (*n* = 8, convenience sample of seven females, one male) was conducted 5 weeks after the intervention. Participants were recruited from survey respondents who consented to being contacted about the focus group during the consent process of the survey. The focus group explored intervention recall, feedback on message framing, intention behind SM engagement, and the use and acceptability of Instagram as a platform for health promotion. The focus group was voice recorded and transcribed verbatim. Thematic content analysis was conducted using the framework approach to guide the identification of themes and subthemes [29–31].

### Statistical analyses

Frequencies of SM metrics collected from each post were used to assess levels of SM engagement. Number of Instagram followers was compared before and after the intervention (% change). Descriptive statistics (frequencies) of survey responses were tabulated for knowledge, behaviour, and attitudes and cross-tabulations used to report responses by sex, age, and other sociodemographic characteristics. Means and standard deviations (where data were normally distributed) were calculated to determine differences in self-reported health behaviours in the pre- and post-intervention survey populations (independent samples). Tests of significance were not conducted due to the feasibility nature of the study and inappropriateness of applying such tests to feasibility studies [32].

## Results

### Population characteristics, knowledge, attitudes, and self-reported behaviour

The pre-intervention survey captured 43 respondents and 41 in the post-intervention survey. Due to variability in the number of responses for different questions (missing data), findings are presented by proportion of respondents. Table 1 presents an overview of the population and Table 2 their SM habits. The majority of respondents were females (88.4% pre, 80.5% post), aged 18–35 years old (95.4% pre, 95.1% post). The population was comprised mostly of post-graduate students (95.4% pre, 92.9% post) living in or near Belfast (69.8% pre, 69.1% post). Survey respondents (86.7% pre-intervention, 85.7% post-intervention) used Instagram frequently or infrequently. As seen in Table 3, the post-intervention respondents reported a better knowledge of PA guidelines (difference of + 12.5%) and higher estimates of weekly and monthly PA (+ 13.7% and + 13.9%). Respondents also appeared to misreport their adherence to PA guidelines, with the proportion of individuals meeting the weekly PA guidelines (11.9% and 25.6%) falling much lower than the proportion who believed they met these guidelines (42.9%, 59.0%)*.* The second survey also captured a higher proportion of respondents who said they ‘always’ look at nutritional labels (+ 10.3%) and had higher daily consumption of 5 + fruit and vegetables (+ 23.3%). However, the second survey also captured higher reported levels of less desirable food-related behaviours. For example, a higher proportion consuming fast food 1–2 days or 3–4 days a week (+ 5.6% and + 2.8%), and meal preparation on 6–7 days a week, was reduced (− 18.0%).

**Table 1:** Characteristics of population captured in survey responses

	Pre-intervention survey *n* (%)	Post-intervention survey *n* (%)
Sex		
°Female	38 (88.4)	33 (80.5)
°Male	5 (11.6)	8 (19.5)
Age		
°18–24	22 (51.2)	18 (43.9)
°25–34	19 (44.2)	21 (51.2)
°35–44	2 (4.7)	2 (4.9)
Student type		
°Undergraduate	0 (0.0)	1 (2.4)
°Postgraduate	41 (95.4)	39 (92.9)
°Not student	2 (4.7)	2 (4.8)
Course type		
°Full time	40 (95.2)	37 (94.9)
°Part time	2 (4.7)	2 (5.1)
Highest qualification		
°Doctorate	3 (7.0)	2 (4.8)
°Master’s degree	18 (41.9)	18 (42.9)
°Bachelor’s degree or equivalent	21 (48.8)	22 (52.4)
°A level or equivalent	1 (2.3)	0 (0.0)
Place of residence		
°Belfast/greater Belfast area	30 (69.8)	29 (69.1)
°Northern Ireland	11 (25.6)	8 (19.1)
°Republic of Ireland	0 (0.0)	1 (2.4)
°Great Britain	2 (4.7)	1 (2.4)
°Other	0 (0.0)	3 (7.1)

**Table 2:** Usage of social media platforms reported by survey respondents (‘pre’, pre-intervention survey; ‘post’, post-intervention survey)

	Twitter		Facebook		YouTube		Instagram		Snapchat		Pinterest		Tumblr		LinkedIn		Google Plus	
	Pre	Post	Pre	Post	Pre	Post	Pre	Post	Pre	Post	Pre	Post	Pre	Post	Pre	Post	Pre	Post
**Not at all**	6 (20.0)	4 (19.1)	1 (3.3)	0 (0.0)	0 (0.0)	2 (9.5)	4 (13.3)	3 (14.3)	9 (30.0)	8 (38.1)	17 (56.7)	13 (61.9)	25 (83.3)	20 (95.2)	11 (36.7)	10 (47.6)	25 (83.3)	18 (85.7)
**Infrequently**	8 (26.7)	7 (33.3)	2 (6.7)	3 (14.3)	11 (36.7)	3 (14.3)	3 (10.0)	5 (23.8)	9 (30.0)	5 (23.8)	11 (36.7)	8 (38.1)	3 (10.0)	0 (0.0)	14 (46.7)	8 (38.1)	5 (16.7)	3 (14.3)
**Frequently**	16 (53.3)	10 (47.6)	27 (90.0)	18 (85.7)	19 (63.3)	16 (76.2)	23 (76.7)	13 (61.9)	12 (40.0)	8 (38.1)	2 (6.7)	0 (0.0)	2 (6.7)	1 (4.8)	5 (16.7)	3 (14.3)	0 (0.0)	0 (0.0)

**Table 3:** Physical activity and nutrition knowledge and behaviour in the pre-intervention and post-intervention periods (*PA*, physical activity)

	Pre-intervention *n* (%)	Post-intervention *n* (%)
Knowledge of PA guidelines (self-reported)		
Yes	26 (61.9)	29 (74.4)
No	16 (38.1)	10 (25.6)
Knowledge of PA guidelines		
Underestimate	13 (31.7)	14 (35.9)
Correct	19 (46.3)	22 (56.4)
Overestimate	9 (22.0)	3 (7.7)
Meeting recommended PA levels (self-reported)		
Yes	18 (42.9)	23 (59.0)
No	24 (57.1)	16 (41.0)
Weekly PA levels		
Lower than recommended MVPA	37 (88.1)	29 (74.4)
Recommended MVPA	2 (4.8)	8 (20.5)
Higher than recommended MVPA	3 (7.1)	2 (5.1)
Monthly PA levels		
Lower than recommended MVPA	36 (85.7)	28 (71.8)
Recommended MVPA	2 (4.8)	10 (25.6)
Higher than recommended MVPA	4 (9.5)	
Look at nutritional levels (frequency)		
Never	2 (4.7)	2 (5.1)
Sometimes	14 (33.3)	16 (41.0)
Most of the time	12 (28.6)	12 (30.8)
Always	14 (33.3)	9 (23.1)
Fruit and veg. consumption (portions per day)		
0	1 (2.4)	0 (0.0)
1–2	19 (45.2)	10 (25.6)
3–4	15 (35.7)	23 (59.0)
5 +	7 (16.7)	6 (15.4)
Fast food consumption (days per week)		
0	15 (36.6)	11 (28.2)
1–2	24 (58.5)	25 (64.1)
3–4	2 (4.9)	3 (7.7)
5 +	0 (0.0)	0 (0.0)
Meal preparation (days per week)		
0–1	2 (4.8)	2 (5.1)
2–3	3 (7.1)	6 (15.4)
4–5	9 (21.4)	12 (30.8)
6–7	28 (66.7)	19 (48.7)

As seen in Table 4, higher importance in the post-intervention period was observed across a number of behaviours, for example ‘having a good night’s sleep’ (+ 3.7%), ‘eating a healthy diet’ (+ 15.8), ‘exercising regularly’ (+ 12.3%), and ‘not drinking alcohol, or drinking only a little’ (+ 11.7%). However, some behaviours, and perceptions of issues related to health and wellbeing, were deemed less important to respondents in the post-intervention period, for example ‘being a non-smoker’ (− 8.4%), ‘having a good figure’ (− 13.2%), ‘not being fat’ (− 9.5%), ‘not being stressed or worried’ (− 13.7%), and ‘knowing about fitness and how to stay fit’ (− 5.5%). Respondents in the second survey were asked if they had consciously made any changes to their approach to PA, diet, or lifestyle in the past month, which some had 44.0%. When asked to detail these changes in free text, the majority was related to PA behaviour (*n* = 12) including walking more due to nicer weather, attending the gym more often, and starting a ‘Couch to 5 K’ programme. Other changes were related to nutrition behaviours (*n* = 9) including eating more fruit and vegetables, cooking their own meals, and joining ‘Slimming World’. Two comments detailed a change in attitudes, one stating they were walking more for mental health and another stated they were allowing themselves to eat more food rather than restricting. One respondent reported negative changes in their behaviour: ‘exam stress has caused me to prioritise studying and exercise less’.

**Table 4:** General wellbeing and lifestyle attitudes in the pre-intervention and post-intervention periods (‘pre’, pre-intervention survey; ‘post’, post-intervention survey)

		See a dentist at least once a year?		Have a doctors’ check-up once a year?		Know about your body and how it works?		Have a good night’s sleep?		Eat a healthy diet?		Be a non-smoker?		Have a good/body figure?	
		Pre	Post	Pre	Post	Pre	Post	Pre	Post	Pre	Post	Pre	Post	Pre	Post
**Not important**	***n* (%)**	5 (11.9)	3 (7.7)	8 (19.1)	7 (18.0)	0 (0.0)	1 (2.6)	1 (2.4)	2 (5.1)	0 (0.0)	0 (0.0)	1 (2.4)	0 (0.0)	2 (4.8)	2 (5.1)
**Of average importance**	***n* (%)**	14 (33.3)	12 (30.8)	15 (35.7)	12 (30.8)	10 (23.8)	8 (20.5)	7 (16.7)	4 (10.3)	12 (28.6)	5 (12.8)	3 (7.1)	7 (18.0)	14 (33.3)	18 (46.2)
**Very important**	***n* (%)**	23 (54.8)	24 (61.5)	19 (45.2)	30 (51.3)	32 (76.2)	30 (76.9)	34 (81.0)	33 (84.6)	30 (71.4)	34 (87.2)	38 (90.5)	32 (82.1)	26 (61.9)	19 (48.7)
		**Exercise regularly?**		**Not be fat?**		**Have friends?**		**Not be stressed or worried?**		**Not drink alcohol or drink only a little?**		**Know about fitness and how to stay fit?**			
		Pre	Post	Pre	Post	Pre	Post	Pre	Post	Pre	Post	Pre	Post		
**Not important**	***n* (%)**	0 (0.0)	1 (2.6)	0 (0.0)	0 (0.0)	0 (0.0)	0 (0.0)	0 (0.0)	0 (0.0)	10 (23.8)	10 (25.6)	3 (7.1)	0 (0.0)		
**Of average importance**	***n* (%)**	17 (40.5)	10 (25.6)	10 (23.8)	13 (33.3)	3 (7.1)	3 (7.7)	5 (11.9)	10 (25.6)	24 (57.1)	17 (43.6)	13 (31.0)	17 (43.6)		
**Very important**	***n* (%)**	25 (59.5)	28 (71.8)	32 (76.2)	26 (66.7)	39 (92.9)	36 (92.3)	37 (88.1)	29 (74.4)	8 (19.1)	12 (30.8)	26 (61.9)	22 (56.4)		

### Engagement with intervention, collecting SM metrics

#### Engagement with SM intervention posts

Table 5 presents SM metrics collected during the intervention. In total, the Instagram posts received 247 ‘likes’ over the 4-week campaign (mean 8.8 per post). The number of likes as a proportion of (original, *n* = 350) followers was 2.5%. Three comments were collected from under two posts (mean 0.11). Four tweets achieved a total of 14 likes (mean 2.25 per post). The three posts on Facebook achieved a total of 51 likes/reactions including 40 likes, eight laugh reactions, and one love reaction (mean 17 per post). In addition, 53 comments were collected from Facebook (mean 17.7 per post).

**Table 5:** Comparison of social media metrics collected across content

	Likes Median (IQR)
Humour	10 (3)
Non-humorous	8 (3)
Physical activity	10.5 (3.75)
Nutrition	7 (2)
General	10 (3)
Substitution	7.5 (2)
Information	10 (3)
Social support	10 (1.5)

Table 6 illustrates difference in engagement across posts. Humorous posts achieved a slightly higher level of engagement (median 10 likes) compared to non-humorous (median 8 likes). PA-related posts and general wellbeing-related posts had the highest engagement (median 10.5 and 10 likes, respectively), whereas nutrition-related posts had a median of seven likes. Information-based posts and social support-based posts had similar levels of engagement (median 10 likes), whereas substitution-based posts had lower engagement (median 7.5 likes). Due to the low number of comments, no comparison was made across the different posts; however, 66.7% (2/3) comments were made under a general wellbeing-related, information-based post, and the other was made under a nutrition-related, substitution-based post.

**Table 6:** Comparison of engagement across social media platforms

	Topic (general/nutrition/physical activity)	Type (information/substitution/social support)	Humorous/non-humorous	Instagram		Twitter		Facebook		
				*Likes, n*	*Comments, n*	*Retweets, n*	*Likes, n*	*Likes/reactions, n*	*Comments, n*	*Shares, n*
Post 1	General	Information	Humorous	10	0	-	-	-	-	-
Post 2	Nutrition	Substitution	Non-humorous	7	0	-	-	-	-	-
Post 3	Physical activity	Information	Non-humorous	12	0	-	-	-	-	-
Post 4	Nutrition	Substitution	Non-humorous	7	0	-	-	-	-	-
Post 5	Physical activity	Information	Humorous	8	0	-	-	-	-	-
Post 6	General	Social support	Non-humorous	10	0	3	9	1	0	0
Post 7	Physical activity	Substitution	Humorous	16	0	1	3	49	30 (53 including replies)	2
Post 8	Physical activity	Information	Non-humorous	15	0	0	1	-	-	-
Post 9	Nutrition	Substitution	Non-humorous	7	0	-	-	-	-	-
Post 10	Nutrition	Substitution	Humorous	8	1	-	-	-	-	-
Post 11	Nutrition	Substitution	Non-humorous	7	0	-	-	-	-	-
Post 12	General	Substitution	Humorous	7	0	-	-	-	-	-
Post 13	Physical activity	Information	Non-humorous	8	0	-	-	-	-	-
Post 14	Nutrition	Substitution	Non-humorous	9	0	-	-	-	-	-
Post 15	Nutrition	Substitution	Non-humorous	10	0	-	-	-	-	-
Post 16	Nutrition	Substitution	Non-humorous	9	0	-	-	-	-	-
Post 17	Nutrition	Substitution	Non-humorous	6	0	-	-	-	-	-
Post 18	Nutrition	Information	Non-humorous	4	0	-	-	-	-	-
Post 19	Nutrition	Substitution	Non-humorous	3	0	-	-	-	-	-
Post 20	Nutrition	Substitution	Non-humorous	8	0	-	-	-	-	-
Post 21	Physical activity	Social support	Non-humorous	8	2	0	1	1	0	0
Post 22	Physical activity	Information	Humorous	11	0	-	-	-	-	-
Post 23	General	Information	Humorous	11	0	-	-	-	-	-
Post 24	Physical activity	Information	Non-humorous	10	0	-	-	-	-	-
Post 25	Physical activity	Social support	Non-humorous	11	0	-	-	-	-	-
Post 26	Nutrition	Substitution	Non-humorous	11	0	-	-	-	-	-
Post 27	Physical activity	Information	Non-humorous	8	0	-	-	-	-	-
Post 28	General	Information	Non-humorous	6	0	-	-	-	-	-

In the 7-week period prior to intervention activities, the number of Instagram followers increased from 323 to 350 (+ 8.4%, 3.86 followers per week). In the 9-week period during which intervention (including survey advertisement) was conducted, Instagram followers increased from 350 to 403 (+ 15.1%, 5.89 followers per week).

#### Intention behind SM engagement

Focus group participants were asked to consider why they would engage with health content on SM. Participants agreed that intention behind ‘liking’ a post depended on the content and the source: ‘Sometimes it’s “aww that’s cute”, or “well done”, or sympathy…’. Participants agreed that engagement depended on the SM platform, and that they ‘like’ more content on Instagram than others, ‘on Instagram, I give out likes freely’. Tagging friends and conversing with the poster were the main motivations behind commenting, ‘I would tag my friends in Facebook posts, and on Instagram we’ll have discussions underneath posts’. The intention behind sharing lays in a willingness to inform others, particularly if something was relevant, interesting, or useful to friends and followers, ‘If it’s relevant, or I feel people would benefit from the information’. Participants also stated that they would occasionally search hashtags and ‘save’ items on Instagram, particularly food-related posts such as recipes.

### Acceptability of the campaign

All focus group participants recalled seeing #WeeStepsToHealth content on Instagram and Facebook, but not on Twitter. Feedback on content was generally positive, and participants preferred posts which provided realistic and modest substitutions to unhealthy behaviours and emphasised wellbeing and mental health benefits of PA and nutrition, ‘moving away from “body image” side of healthy eating and exercise, and focusing on well-being and mental health, which is a refreshing change’. However, some participants did not like the focus on calories and believed that content looked targeted towards women, ‘Including calories is risky for some people who are at risk of eating disorders.’

Three key themes were identified from the focus group: humour for engagement, importance of a reliable source, and signposting. Participants believed that the use of humour in campaign posts caught their attention, encouraged them to read the content, and improved recall, ‘I’m more likely to remember the funny posts’. Participants trusted the campaign content in large part because they trusted the source (i.e. Queen’s Graduate School platform), ‘being posted by Queen’s Graduate School meant I trusted it… I see it as a reputable institution…’. Finally, particularly within the context of humorous posts, signposting was identified as a positive element within the campaign, that is, providing links to external places where the audience can visit for more information, ‘I really like the use of a link, it’s helpful to be signposted to a reputable source…’.

The consensus was that the campaign provided a refreshing change from content on Instagram, appeared professionally sourced and presented, and acted as a useful daily reminder of health. ‘They’re good as reminders to be slightly healthier in your everyday life’. Participants felt comfortable being targeted by a health campaign on SM, because they were aware large corporations on SM are targeting them and believed a campaign such as this provides more value, ‘We know we’re targeted by ads, stuff that is factually based is a more useful than someone selling pills’. Participants deemed Instagram an acceptable platform for health promotion. Furthermore, participants felt that there is a need for positive, evidence-based messaging among the content often seen on Instagram, ‘It’s nice to see a focus more on “wellbeing” and “healthy bodies”, rather than “skinny” and “losing weight”’.

## Discussion

### Principal findings

The aim of this study was to investigate the feasibility and acceptability of using Instagram to engage post-graduate students in a mass communication SM-based health intervention. Post-intervention survey respondents had higher levels of PA, fruit, and vegetable consumption and paid more attention to nutritional labels. Intervention content achieved positive engagement from followers, an increase in the growth rate of followers for the account, and humorous messages received a higher level of engagement on Instagram than non-humorous content. Participants in the post-intervention focus group liked the campaign, trusted the information source, and believed Instagram to be an appropriate platform for delivering the content. Focus group participants believed SM engagement to be circumstantial, depending on platform and poster. Findings indicate that Instagram is a feasible and acceptable platform for engaging post-graduate students in a SM-based mass communication health intervention, and that humour has the potential to encourage further engagement in this population.

### Social media engagement

Overall, engagement with the Instagram posts was moderately positive. When engagement metrics are presented as a proportion of followers, it is clear to see that Instagram posts were much more likely to receive likes (2.5%) than comments (0.03%). This is a trend noted in previous mass communication interventions [33, 34] and may be due to a number of reasons that were discussed in the focus group. For example, to like a post is a simpler way to engage, individuals may not have any desire to communicate directly with The Graduate School, and users may be conscious of their SM persona (i.e. that which they are presenting to followers). This has implications for SM-based health interventions, in that two-way communication with audiences may be limited if relying on a publicly visible comment section for input from followers.

### Humorous content

Humorous posts achieved a moderately higher level of engagement (median 10 likes) compared to non-humorous posts (median 8 likes). This is commensurate with feedback from focus group participants, who said they were more likely to stop and read funny posts. Previous research has identified similar trends in other mass communication health interventions [19, 20]. Tying in to humour, edutainment (entertainment-education) is used increasingly in supplementing and complementing public health interventions and has shown promise in health promotion for young people [21, 35]. Whilst humorous posts may receive a higher level of engagement, it may be more difficult to convey a clear health message. Focus group participants stated that they liked a funny post where they could swipe to find out more information. It is therefore clear that humour alone is not enough to encourage a change in knowledge, attitude, and behaviour in these areas but must be anchored with information regarding PA, nutrition, and general wellbeing issues.

### Acceptability of campaign

Findings echo previous studies which suggest that young adults in this population are open and willing to receive health information via SM [14]. Instagram sometimes hosts ‘unhealthy’ images and promotion of unhealthy behaviours [3, 5]. This sentiment was reflected among focus group participants, who felt this intervention was a refreshing change to the norm on Instagram which tends to focus on ‘skinny bodies’ and weight loss. The public health community must acknowledge the importance, and danger, of visual platforms such as Instagram, in how unhealthy behaviours and attitudes can spread. Future health interventions should consider Instagram as a dissemination platform, particularly in this population. Furthermore, focus group participants found the source of the information (Queen’s Graduate School) to be a reliable source they were likely to trust, highlighting the potential of ‘information intermediaries’ (i.e. non-health-related accounts which are independently reputable) in future health interventions on SM. However, due to the unique characteristics of the post-graduate student population captured (i.e. highly educated young adults), issues arise in whether these findings are applicable to other populations, particularly older populations.

### Feasibility of data collection methods and study design

Whilst SM metrics were readily available measures of SM engagement, they may not accurately reflect true engagement with the intervention [36]. There is no guarantee of authenticity of the SM metrics collected (e.g. risk of fake accounts or bots), meaning we cannot say with certainty that each like represents an individual in the population [37]. Additionally, a degree of interpretation is required regarding the intention behind metrics. Facebook proved more useful in this regard with the availability of a variety of ‘reactions’, which are harder to misinterpret in terms of sentiment and expression, for example a ‘haha’ reaction on Facebook likely implies amusement, whereas a ‘like’ on Instagram may imply amusement but is unclear. However, the survey and focus group participants helped contextualise intentions behind SM metrics, which helped contextualise engagement. Researchers must therefore consider the balance between successful content dissemination on SM and meaningful data collection, meaning the collection of likes and other SM metrics may not yet be sufficient in providing true indications of engagement with intervention content.

Finally, with regards the study design, there were a number of external factors which could have influenced the findings. For example the timing of the campaign overall, but specifically, the post-intervention survey as it was conducted close to exam period for post-graduate students. In addition, the lack of control group, and two independent samples captured by the surveys, makes it difficult to conclude on the effectiveness of the intervention. Future studies should therefore consider the following: (i) the timing of the intervention within the university semester (e.g. choosing an intervention timeframe outside of exam season); (ii) implementation of a control group (for example a sample of post-graduate students from a different university, unexposed to the SM messages but assessed according to the same outcomes); and (iii) the recruitment of the same sample for completion of pre- and post-intervention surveys assessing behavioural outcomes (e.g. by verifying identity of surveyrespondents).

### Strengths and limitations

There are a number of strengths of this study: (i) the intervention was developed using the MRC framework for developing and evaluating complex interventions, (ii) the study is novel, (iii) content was created with input from the target population, and (iv) the target population provided context for the measures collected (i.e. SM metrics). However, a number of limitations were identified: (i) content was posted alongside regular content on the Queen’s Graduate School Instagram account, limiting the ability to state with confidence that increased followership was due to the intervention (however, this does reflect how organisational platforms provide their content); (ii) a relatively small level of engagement across the intervention means conclusions regarding message frame are preliminary; (iii) the nature of capturing survey respondents through SM (followers of the Instagram account) meant that a small number of individuals outside the population were captured (e.g. 1 undergraduate student was captured in the post-intervention survey); (iv) ethnicity and socioeconomic data was not collected and thus impact of ethnicity or socioeconomic status on the findings cannot be discussed; and (v) the pre- and post-intervention surveys potentially captured two independent samples, making comparison in the pre and post stage difficult.

## Conclusion

Instagram was an acceptable platform for engaging post-graduate students in a mass communication social media-based health intervention. Additionally, humour encouraged moderately higher SM engagement, and qualitative findings made clear the importance of disseminating information from a reliable account online. The intervention demonstrated potential feasibility and acceptability and should be further tested in a pilot study among a postgraduate student population following MRC guidance.

## Supplementary information

**Additional file 1.**

**Additional file 2.**

## Data Availability

The datasets used and/or analysed during the current study are available from the corresponding author on reasonable request.

## Abbreviations

BCT: Behaviour change technique
CHERRIES: Checklist for Reporting Results of Internet E-Surveys
MRC: Medical Research Council
QUB: Queen’s University Belfast
PA: Physical activity
SM: Social media
