# Supplementary material for: Investigating the feasibility and acceptability of using Instagram to engage post-graduate students in a mass communication social media-based health intervention, #WeeStepsToHealth
Source: Pilot Feasibility Stud. 2022 Dec 12;8:254. doi: 10.1186/s40814-022-01207-9 (PMC9743718; doi:10.1186/s40814-022-01207-9)
Supplement: Supplementary file 1 — Additional file 1. [file 40814_2022_1207_MOESM1_ESM.docx]

**Supplementary file 1: #WeeStepsToHealth Instagram posts**

| Post no. | Image(s) | Caption |
| --- | --- | --- |
| 1 | 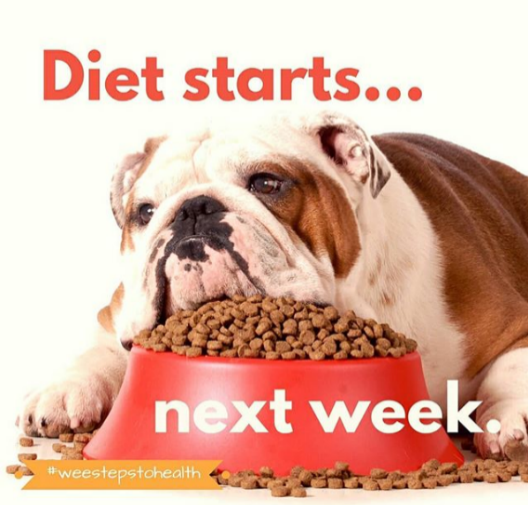  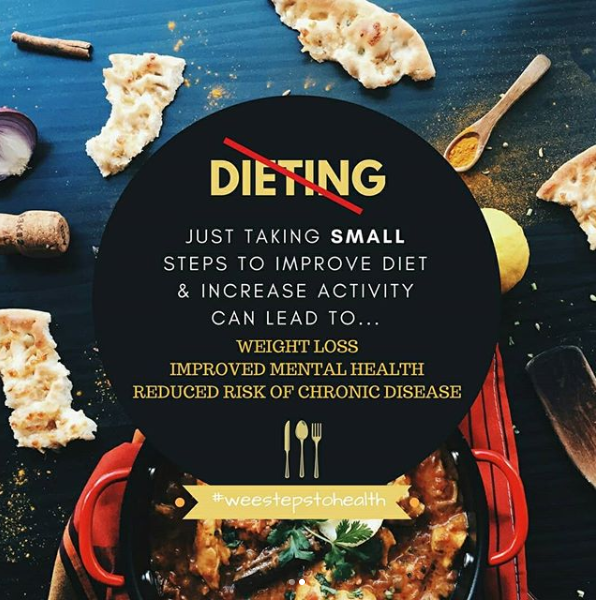 | Keep an eye on #WeeStepsToHealth over the next few weeks as there will be a series of posts aimed at helping you to make small changes to improve your overall wellbeing! |
| 2 | 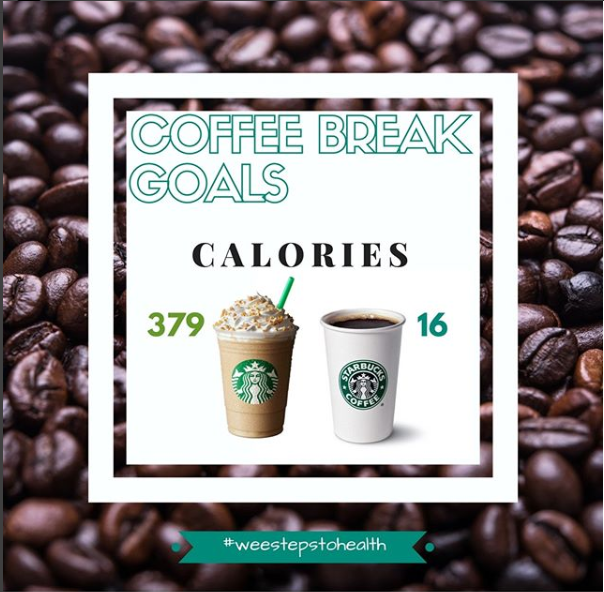 | Do you know what you’re drinking?  A grande caramel frappuccino is not only high in calories but 60% of the recommended daily sugar intake for the average adult.  Consider swapping your high calorie, sugary coffee for a lower calorie, less sugary option #WeeStepsToHealth  *CALORIE BREAKDOWN*  *Starbucks Caramel Frappuccino (grande) = 379 kcal*  *Cappuccino, skimmed milk (grande) = 103 kcal*  *Americano, no milk (grande) = 16kcal* |
| 3 | 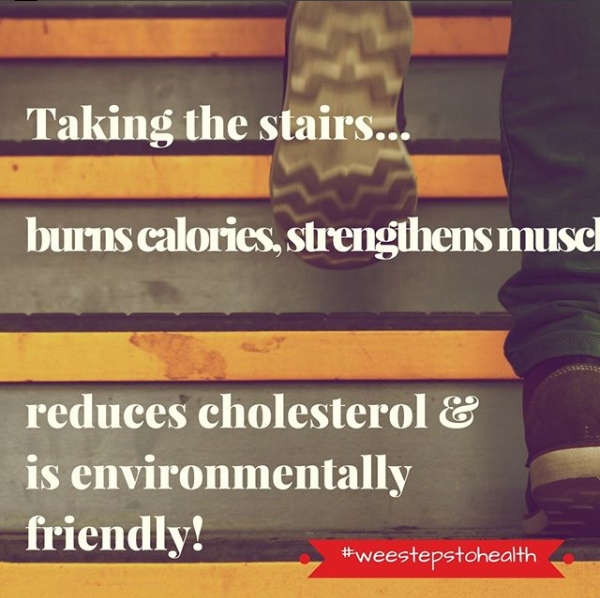 | Being more active doesn’t mean you need to go to the gym every day.  Making small changes to your day, such as taking the stairs rather than the lift or escalators, is an easy way of being more active and also kinder for the environment. |
| 4 | 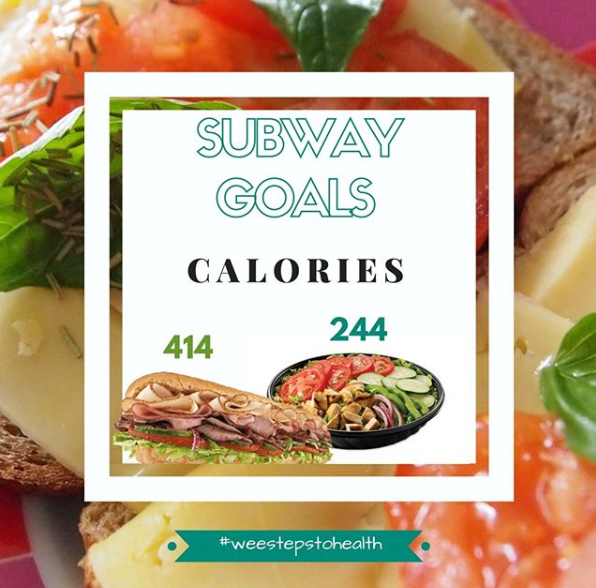 | Switching to a salad version of your favourite sandwich is one easy way of cutting down lunchtime calories #WeeStepsToHealth  *CALORIE BREAKDOWN*  *Before cheese or sauces, 9 grain bread 6 inch; based on cucumber, lettuce, olives, onions, peppers, tomato*  *Source http://www.subway.com/en-gb/menunutrition/* |
| 5 | 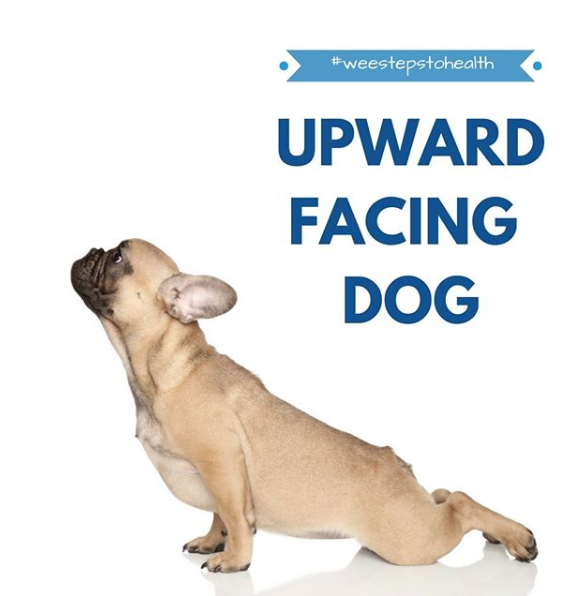 | If you’re looking for a low-resistance exercise to help clear your mind, yoga might be just the thing you’re looking for. You don’t even have to go to a class, there are tons of free yoga tutorials on YouTube.  See link in bio for a 20min introductory session on YouTube  #WeeStepsToHealth |
| 6 | 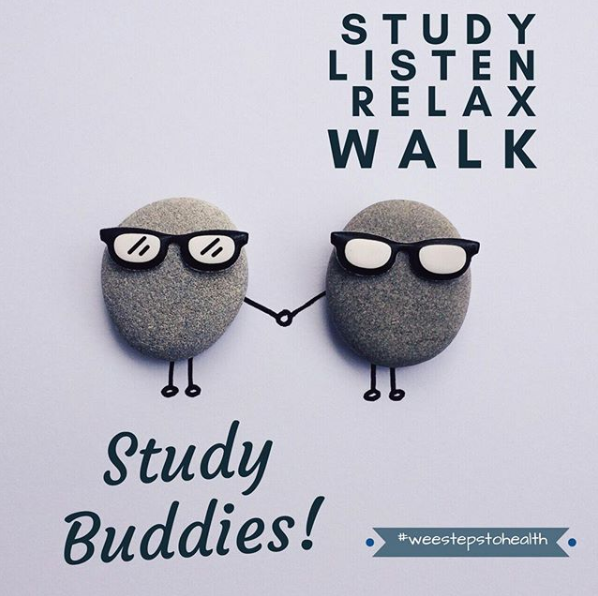 | Get yourself a study buddy who cares about more than just results; take some time to take care of each other.  Did you know walking has been scientifically proven to improve your mood?  Tag your study buddy to invite them for a study break walk!  #WeeStepsToHealth |
| 7 | 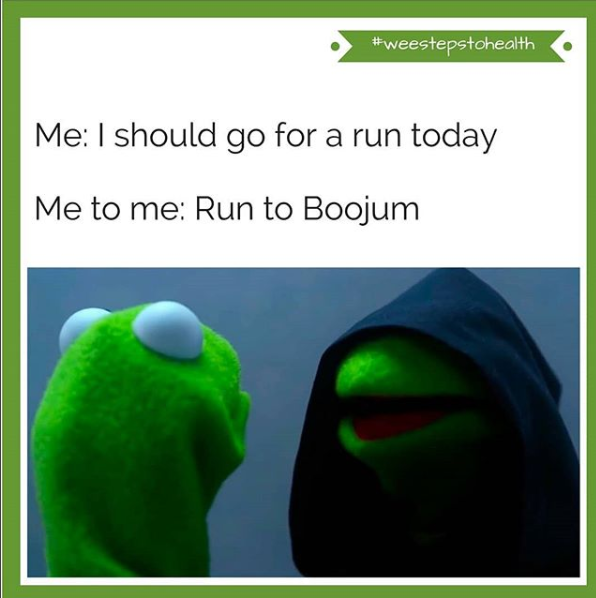  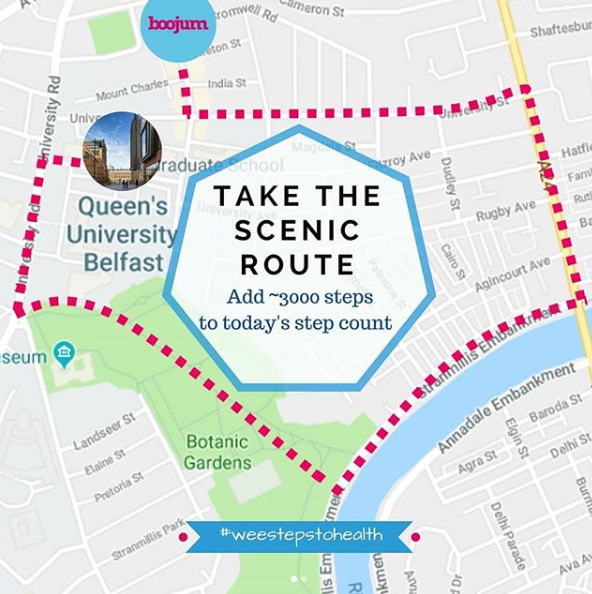 | A run to Boojum is better than no run at all.  Switch up your lunchtime routine to increase your step count for the day, and make that Boojum taste a little less guilty!  #WeeStepsToHealth |
| 8 | 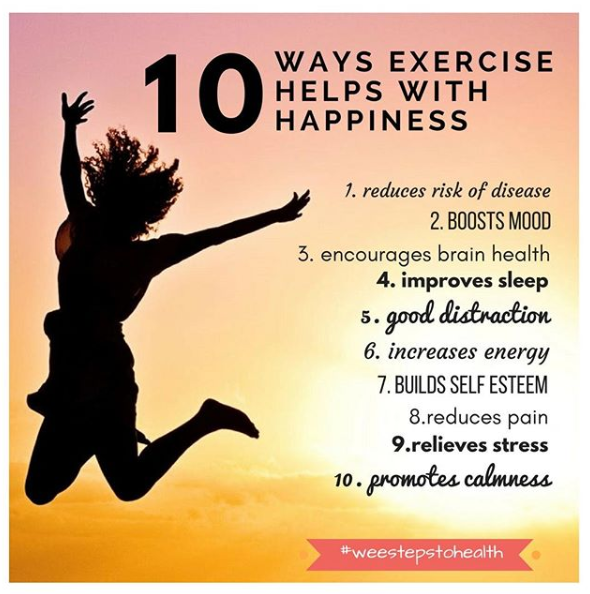 | Exercise is good for more than just your body!  Especially with deadlines and exams looming, exercise can help improve your mental well-being and help you live your best life!  #WeeStepsToHealth  #WellbeingWednesday  #HappyHumpDay  10 Tips taken from ParticipACTION |
| 9 | 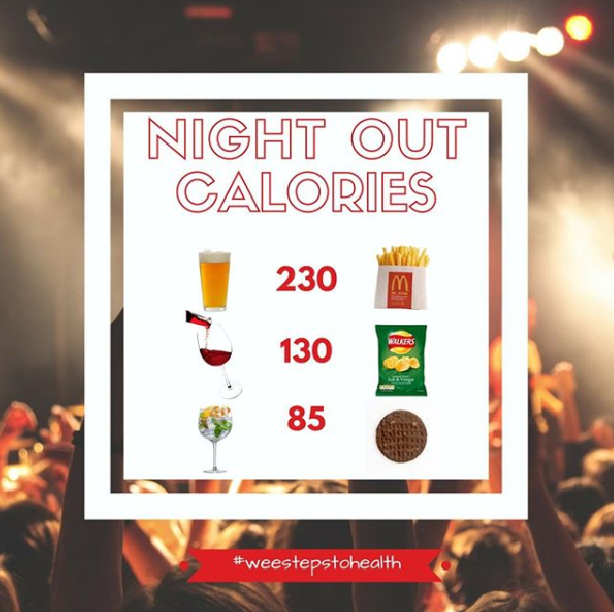 | Just because you’re not chewing doesn’t mean there’s no calories.  You don’t have to cut alcohol out of your diet if you’re reducing your calorie intake, but moderation is key  #WeeStepsToHealth |
| 10 | 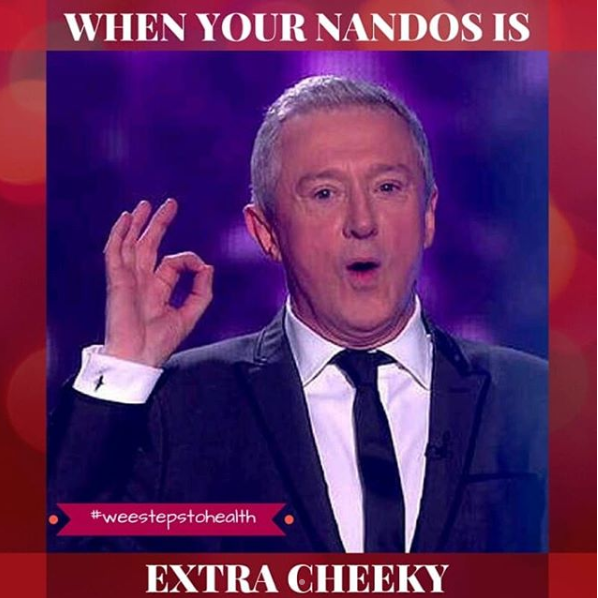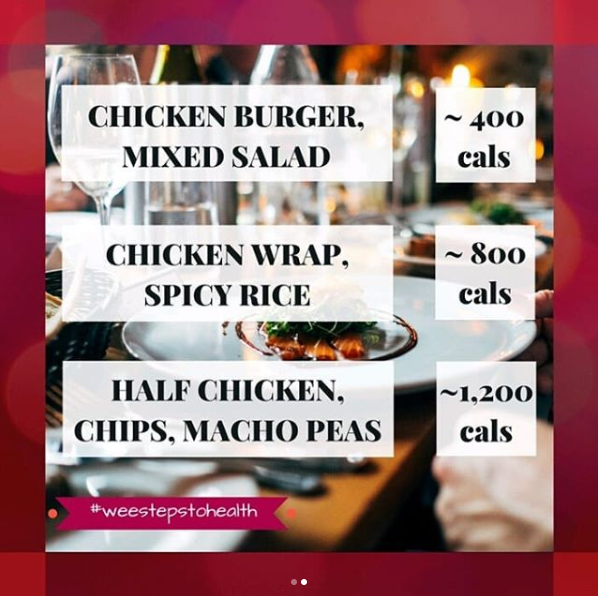 | Just how cheeky is your Nandos? Turns out it’s up to you! A trip to Nandos doesn’t always need to be cheeky, making smarter choices can help to reduce the calorie count when eating out #WeeStepsToHealth |
| 11 | 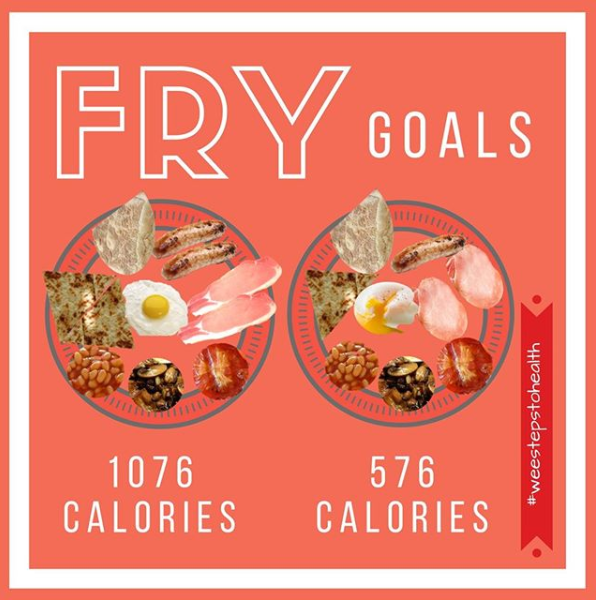 | There’s little better than starting your Saturday with a fry up, but does your fry have 1000+ calories like the one above?  Making small changes to your fry such as…  i) one less sausage and only half a potato farl  ii) bacon medallions instead of rashers  iii) poached egg instead of fried  iv) grilling, instead of frying, mushrooms and tomatoes  …can almost half the calorie count! Same ingredients, less guilt. #WeeStepsToHealth  CALORIE BREAKDOWN  168Kcal per sausage (Denny Pork Sausages 56.8g)  Smoked bacon rashers 102kcal per 2 rashers (Sainsbury's Smoked Back Bacon)  Smoked bacon medallions 62kcal per 2 medallions (Sainsbury's Smoked Bacon Medallions, Be Good To Yourself)  110kcal per potato bread farl (Rankin)  110kcal per 0.5 soda bread farl (Ormo)  78kcal per 100g Heinz baked beans  Fried tomato large 130 kcal  Grilled tomato large 30 kcal  Fried mushrooms 0.5 cup 120kcal  Grilled mushrooms 0.5 cup 8 kcal  Fried egg 90kcal  Poached egg 65kcal |
| 12 | 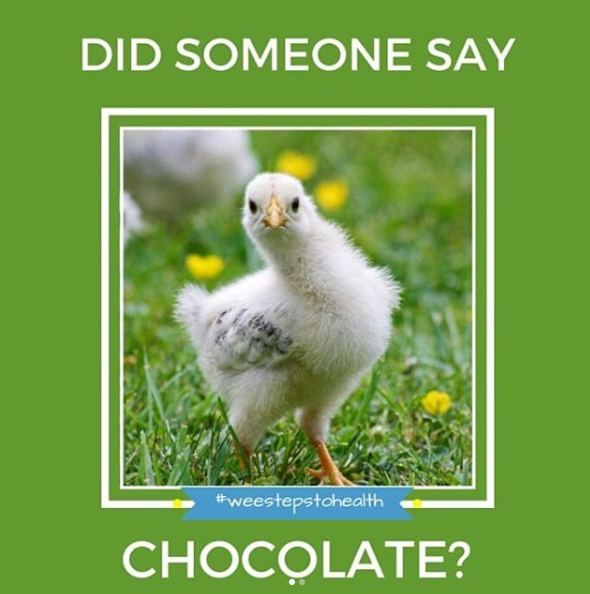  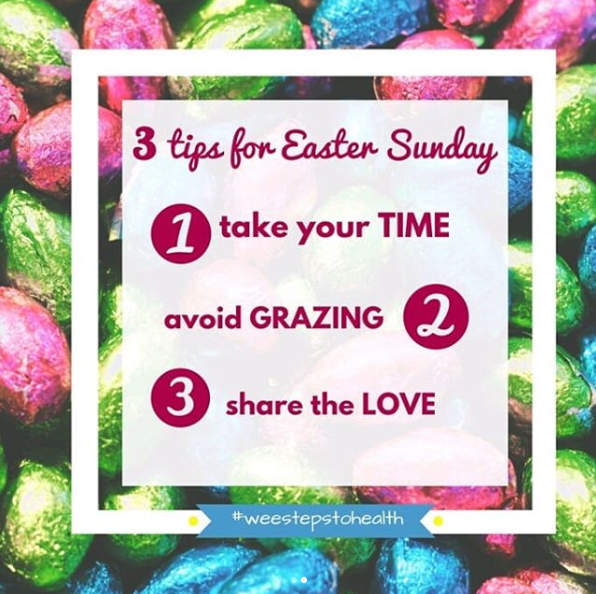 | Tips for not overdoing it on Easter Sunday:  1. Take your time, not all of your chocolate has to be eaten today.  2. Avoid grazing all day, this makes it harder to track exactly how much you’ve eaten.  3. Show those you love that you care by sharing your egg with them!  #WeeStepsToHealth |
| 13 | 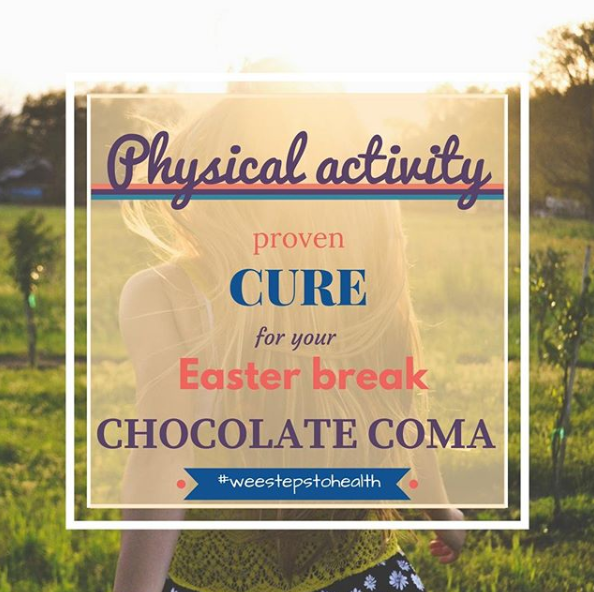 | Too much chocolate in your belly after yesterday?  Shake yourself out of your chocolate coma and make the most of the longer nights - grab some family or friends and go for a long walk!  #WeeStepsToHealth |
| 14 | 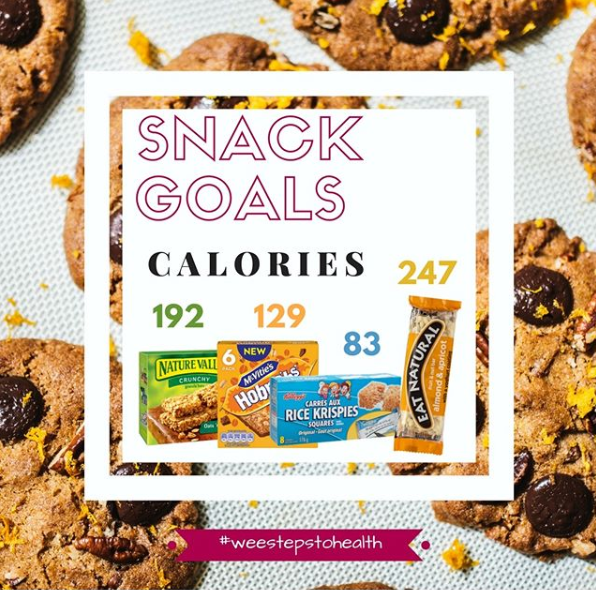 | These little bars are so handy for on-the-go snacking, but do you know how many calories are in your snack bar of choice? They may all look the same size but snack bars range from low calorie/small portion size to high calorie, large portion size #WeeStepsToHealth |
| 15 | 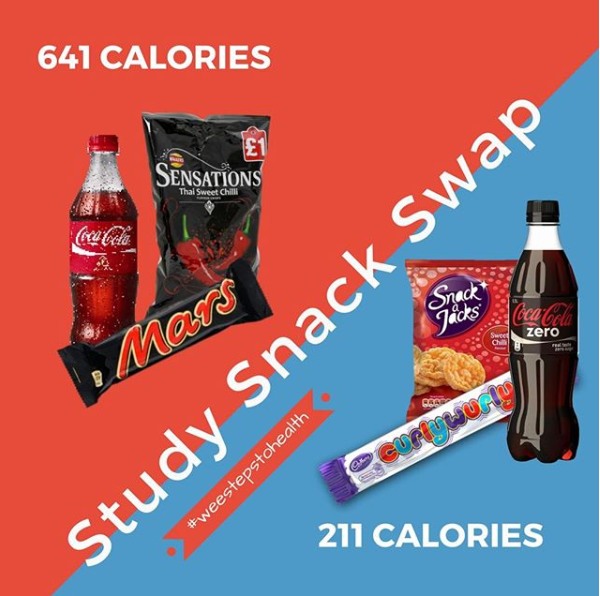 | Did you know that you can drastically reduce your study snack calorie intake by replacing full sugar drinks with zero sugar drinks and choosing smaller portioned, lower calorie snacks. #WeeStepsToHealth  #workingwednesday  #wednesdaywellbeing  #studysnack  #weestepstohealth  *CALORIE BREAKDOWN*  *500ml coca cola (210kcal), 40g Thai Sweet Chilli Sensations (201kcal), 51g Mars bar (230kcal) = 641 calories*  *500ml coke zero (2kcal), 22g Snack a Jack Sweet Chilli (91kcal), 26g Curly Wurly bar (118kcal) = 211 calories* |
| 16 | 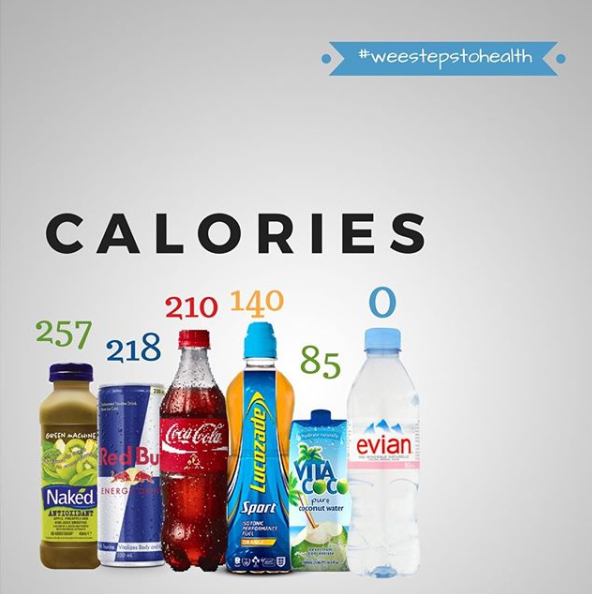 | When it comes to drinks, nothing beats water; zero calories and zero sugar no matter how much you drink, and it’s free! Consider swapping out your high calorie, high sugar drinks for water  #WeeStepsToHealth |
| 17 | 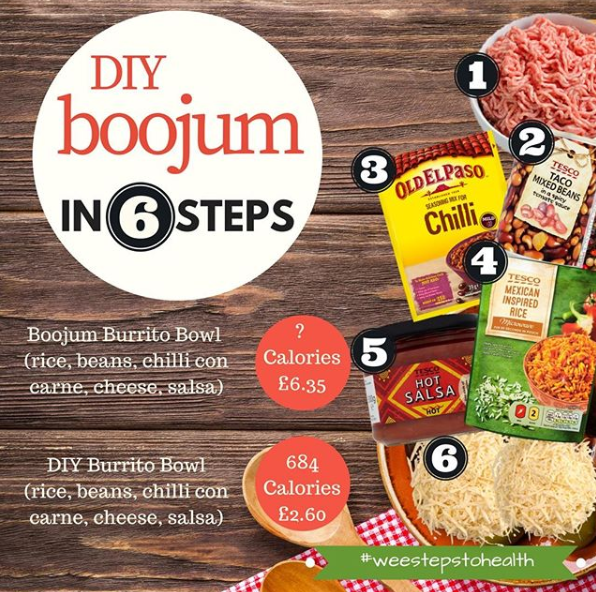 | Working hard to get your Boojum t-shirt? It’ll only cost you over £200! Preparing your own meals means easier i) portion control ii) cost control and iii) knowing exactly how many calories you’re putting away. Consider changing your takeaway #Friday to a #fakeaway Friday #WeeStepsToHealth |
| 18 | 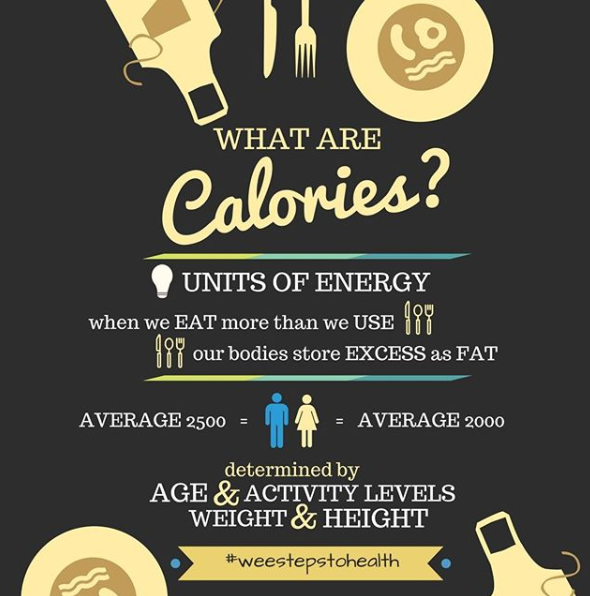 | What are calories anyway? Do you know how many calories you should be eating a day? Follow the link in our bio to calculate how many calories you should be consuming a day for i) maintenance ii) weight loss.  #WeeStepsToHealth  #SaturdaySums  #SaturdayKitchen |
| 19 | 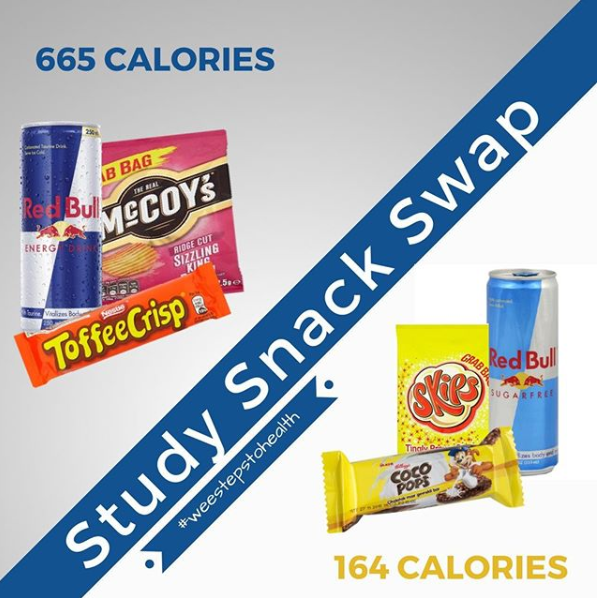 | The study snack on the left is 4 times as many calories as that on the right – would you make the swap?  You can drastically reduce your study snack calorie intake by replacing full sugar drinks with zero sugar drinks and choosing smaller portioned, lower calorie snacks. #WeeStepsToHealth  *CALORIE BREAKDOWN*  *500ml Red bull = 218 kcal, McCoys = 249cal (47.5g), Toffee crisp = 198kcal (38g)*  *500ml Red Bull Zero = 9 kcal, Skips = 71kcal (13.1g), Cocopops snack bar = 84 kcal (20g)*  #sundaystudy  #studysnack  #snapswap  #sweetsunday |
| 20 | 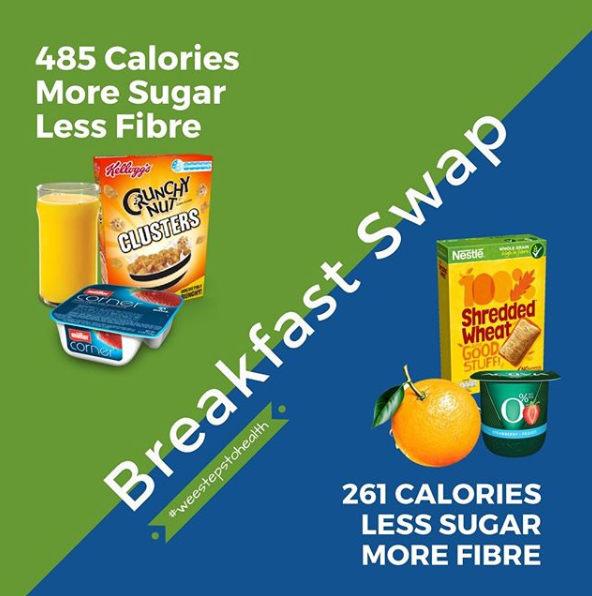 | Breakfast is an important meal to set the tone for the day, but are you paying attention to your morning sugar, fibre and calorie intake? Fill yourself for less by choosing cereals high in fibre, switching sugary yoghurts for lower sugar yoghurts and swapping fruit juice for fruit #WeeStepsToHealth  *CALORIE BREAKDOWN*  *250ml Tropicana = 108kcal, Muller corner strawberry 150g = 176kcal, Crunchy Nut Honey & Nut Clusters 45g = 201kcal, 1.8g fibre*  *100g orange = 36kcal, Activia 0% 125g = 63kcal, Shredded Wheat 45g = 162kcal, 12g fibre*  #mondaymotivation  #goodmorning  #mondaymeal |
| 21 | 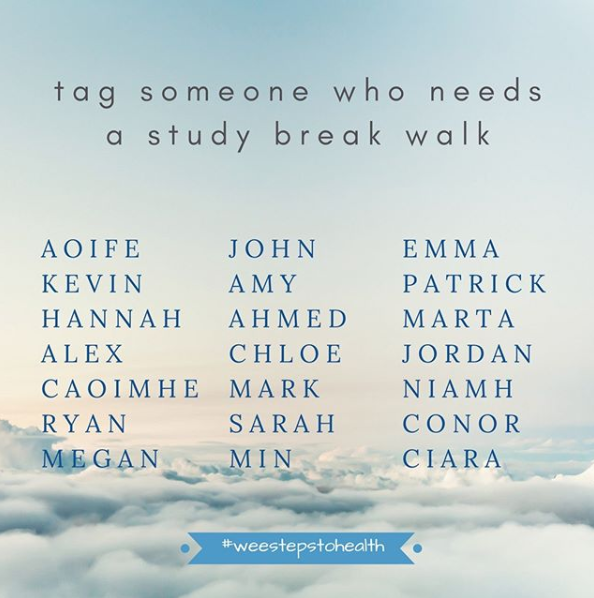 | Tag a friend below that desperately needs a break from the books!  #WeeStepsToHealth  #tagafriend  #takeabreak  #hitrefresh  #pglife |
| 22 | 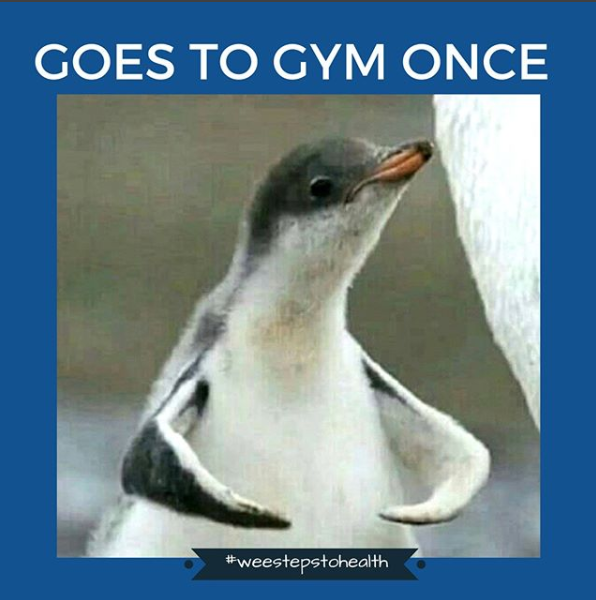 | You may not see instant results from exercising but you may see an instant shift in your mood.  Not only do you improve your physical health when you’re more active, but you’re improving your mental health. Those who get regular physical activity have up to 30% lower risk of depression!  #WeeStepsToHealth |
| 23 | 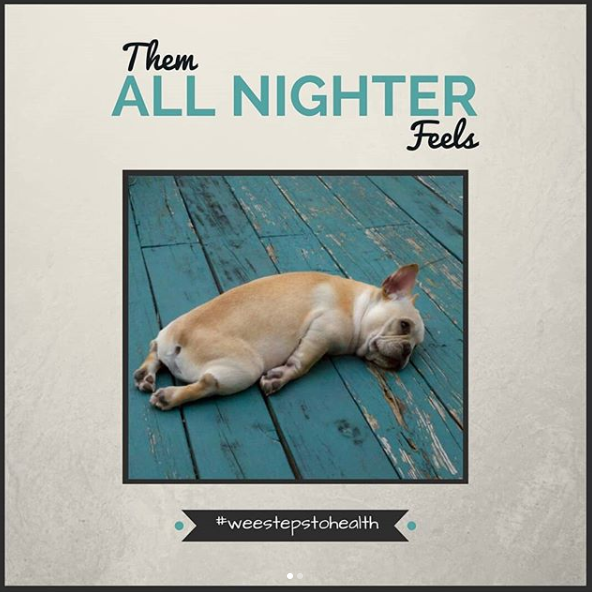  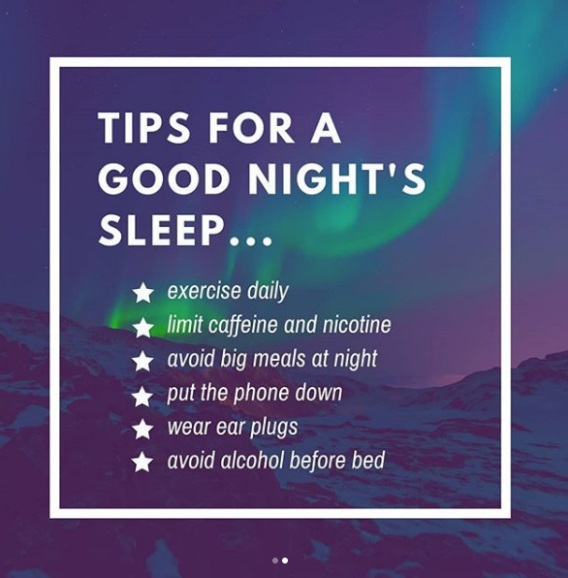 | All nighters cramming in the library might seem like a good idea, but a good night’s sleep may actually help you more with that assignment. Memory recall and ability to maintain concentration are improved when an individual is rested, and you’ll feel much better for it #WeeStepsToHealth |
| 24 | 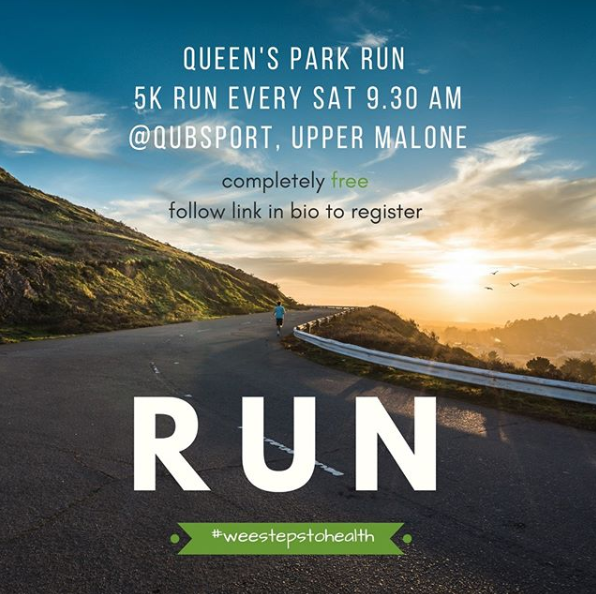 | Start your weekend off right with a free 5k park run right here at Queen’s. The Queen’s park run is completely free to enter but you need to register – follow link in bio for more details and registration #WeeStepsToHealth |
| 25 | 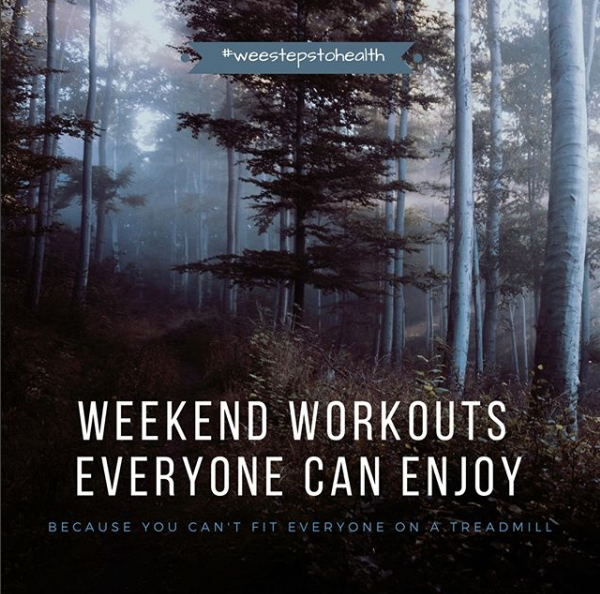 | Just because you’re not in the gym, doesn’t mean you’re not exercising. Try something different this weekend, and take advantage of the amazing and unique activities across NI #WeeStepsToHealth |
| 26 | 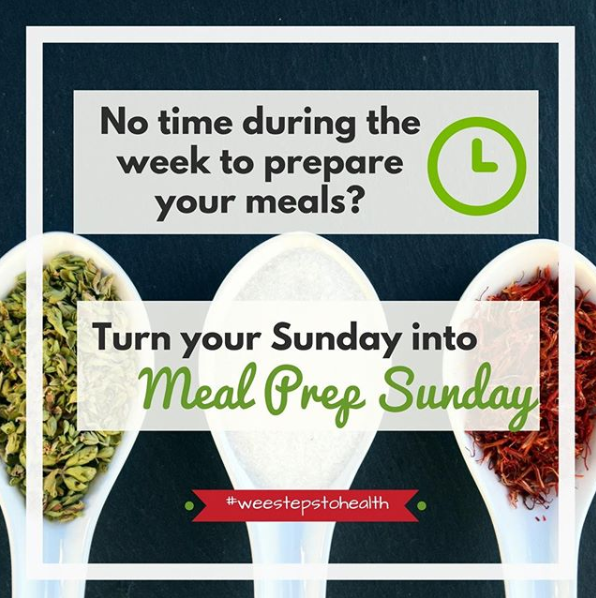 | Is time a factor in stopping you from eating well during the week? Make today a meal prep day:  1) cook a big batch of food  2) separate portions into tupperware  3) keep in the fridge  4) reheat throughout the week  #WeeStepsToHealth |
| 27 | 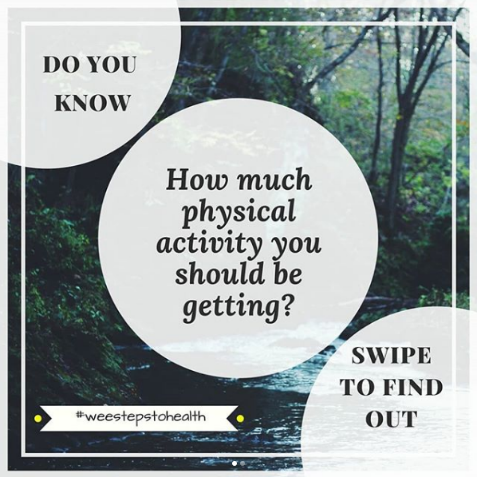  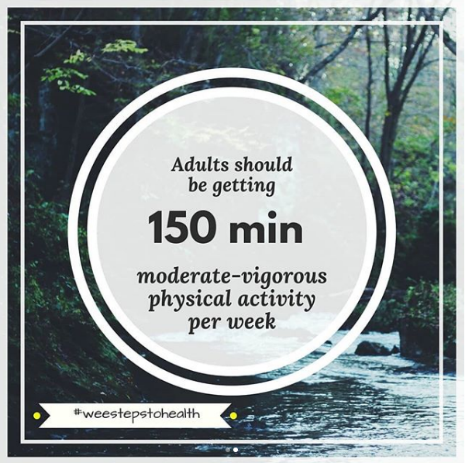 | The guidelines for healthy adults recommend getting at least 150 minutes of moderate to vigorous activity a week OR 75 minutes of vigorous aerobic activity. Activity can be achieved in whichever way you prefer; whether it is brisk walking, household chores or playing football. #WeeStepsToHealth |
| 28 | 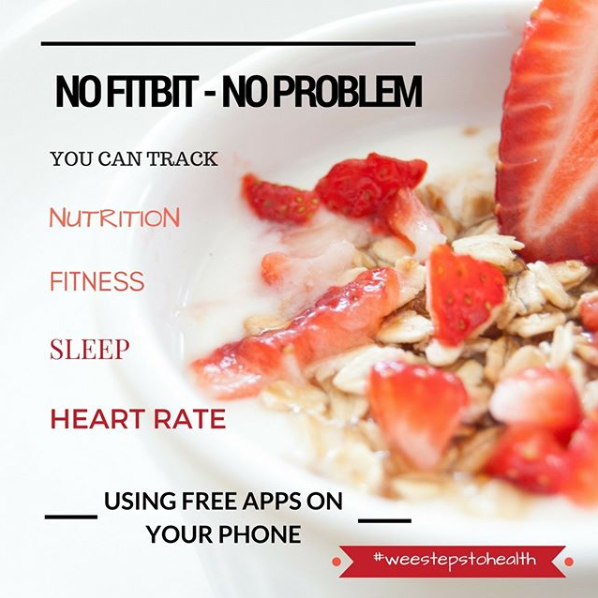 | Interested in tracking your nutrition and fitness but don’t fancy the Fitbit price tag? Search your app store for free health apps to get you started #WeeStepsToHealth |
