# Supplementary material for: Investigating the feasibility and acceptability of using Instagram to engage post-graduate students in a mass communication social media-based health intervention, #WeeStepsToHealth
Source: Pilot Feasibility Stud. 2022 Dec 12;8:254. doi: 10.1186/s40814-022-01207-9 (PMC9743718; doi:10.1186/s40814-022-01207-9)
Supplement: Supplementary file 2 — Additional file 2. [file 40814_2022_1207_MOESM2_ESM.docx]

**Survey: “Wee Steps to Health” health promotion campaign**

You are invited to take part in a research study which is investigating knowledge and attitudes regarding physical activity and diet behaviours. This study is being conducted by researchers from the Centre for Public Health, Queen’s University Belfast in collaboration with The Graduate School, Queen’s University Belfast.

This questionnaire asks you a series of questions about you and regarding your knowledge of the guidelines and attitude towards physical activity and nutrition. Your participation in this survey is completely anonymous and responses are confidential.

By completing this survey you are confirming that:

- you are aged 18 years or older;
- you understand that your participation is voluntary and that you are free to withdraw at any time;
- you understand all data will be treated securely as described by Data Protection and stored appropriately as required by the University;
- you understand that you will not be identifiable in any data published in relation to this project;
- you agree to participate in the “Wee Steps to Health” Internet Survey.

There are 4 sections in the questionnaire. Please answer all questions in each section as instructed. However, if you do not wish to answer a question(s) in any section just leave the response blank. The questionnaire should take 10-15 minutes to complete.

1. **Socio-demographic Module**
2. Gender:

Male
Female
Other: ________________________­­­
Prefer not to say

1. Age: (Respondents must be aged 18 or older):

18-25
25-35
35-45
45-55
55+

1. Student type:

Undergraduate
Postgraduate
Not a student

1. Student status:

Full-time
Part-time
Other

1. What is your highest qualification?

Doctorate
Master’s degree
Bachelor’s degree or equivalent

FE college degree, diploma, certificate HND, HNC

FE higher diploma, certificate HND, HNC

FE ordinary diploma, certificate OND, ONC

A-Levels or advanced GNVQ/NVQ Level 3 / Advanced GNVQ, BTEC National Diploma

5+ GCSE passes / NVQ Level 2 / Intermediate GNVQ / O'level/ CSE Grade 1 / senior certificate

1-4 GCSE passes / NVQ Level 1 / Foundation GNVQ / O'level / CSE grade 1 / Junior certificate

Trade apprenticeship

RSA / CSE

None

Other - please specify

1. Is your current place of residence:

Student accommodation
Rented privately
Owned outright
Being bought with mortgage/loan
Parental/guardian home
Other, please specify: ________________________

1. Do you currently live in:

Belfast/greater Belfast area

Northern Ireland

Republic of Ireland

Great Britain

Europe, please specify: ________________________

Other, please specify: ________________________

1. **Physical activity knowledge, attitudes and behaviour module**
2. Are you aware of the current guidelines for physical activity for adults?

Yes

No

1. Please select which of the following you believe to be the current guidelines for physical activity for adults *per week*:

*moderate activity = requires moderate effort; noticeably raised heart rate
*vigorous activity = requires large amount effort; rapid breathing, substantial increase in heart rate

- 1. 50 minutes of vigorous intensity
  2. 100 minutes of vigorous intensity
  3. 100 minutes of moderate-vigorous intensity
  4. 150 minutes of moderate-vigorous intensity
  5. 200 minutes of moderate-vigorous intensity

1. Do you think you are achieving these recommended activity levels?

Yes

No

1. “In the past week, on how many days have you done a total of 30 minutes or more of physical activity, which was enough to raise your breathing rate. This may include sport, exercise, and brisk walking or cycling for recreation or to get to and from places, but should not include housework or physical activity that may be part of your job”

Number of days: (0-7)
________________________

1. “In the past month, on how many days have you done a total of 30 minutes or more of physical activity, which was enough to raise your breathing rate.

Number of days: 0-31
________________________

1. **Nutrition knowledge, attitudes and behaviour module**
2. How often do you look at nutritional labels on the food/drinks you consume?

Always

Most of the time

Sometimes

Never

1. How many portions of fruit/veg would you consume on an average day?

0

1-2

3-5

5+

1. In the last 7 days, on how many days did you consume fast food?

______ days/week

1. Please give specific examples of these fast food establishments:

________________________

| 1. **.    How often do you eat or drink any of the following?** *(Please tick ()****one****box for each row).* | **More than once a day** | **Once a day** | **3-6 days a week** | **1-2 days a week** | **less than weekly** | **Never** |
| --- | --- | --- | --- | --- | --- | --- |
| Sweets, chocolate bars or biscuits (including wrapped chocolate biscuits like Twix or KitKat) |  |  |  |  |  |  |
| Buns, cakes or pastries or desserts |  |  |  |  |  |  |
| Fizzy drinks or squashes that contain sugar (e.g. coca cola, Ribena, Club Orange) |  |  |  |  |  |  |
| Diet drinks (e.g. Diet Coke, Sprite Zero, Diet Club) |  |  |  |  |  |  |

1. In the last 7 days, on how many days did you prepare your own meals?

______ days/week

1. Please select, if any, deterrents to preparing your own meals:

Someone else (parent, spouse, housemate) cooks for me

Lack of time

Too expensive

I don’t know how

Too messy

Other, please specify: ________________________

1. In the last 7 days, on how many days did you consume alcohol?

______ days/week

1. How many drinks would you have on one of those days?

1-2/3-4/5+

1. Please give specific examples of alcoholic drinks consumed:

________________________

1. In YOUR OPINION how important is it for YOU to:

Tick only one box for each section

|  | Very  Important | Of average  Importance | Not  Important |
| --- | --- | --- | --- |
| See a dentist at least once a year? | 1 | 2 | 3 |
| Have a doctors’ check up once a year? |  |  |  |
| Know about your body and how it works? |  |  |  |
| Have a good night’s sleep? |  |  |  |
| Eat a healthy diet? |  |  |  |
| Be a non-smoker? |  |  |  |
| Have a good/body figure? |  |  |  |
| Exercise regularly? |  |  |  |
| Not be fat? |  |  |  |
| Have friends? |  |  |  |
| Not be stressed or worried? |  |  |  |
| Not drink alcohol, or drink only a little? |  |  |  |
| Know about fitness and how to stay fit? |  |  |  |

1. **Social media module**
2. How often do you use the following social media platforms:

|  | Not at all | Infrequently | Frequently |
| --- | --- | --- | --- |
| Twitter |  |  |  |
| Facebook |  |  |  |
| Pinterest |  |  |  |
| Tumblr |  |  |  |
| Instagram |  |  |  |
| LinkedIn |  |  |  |
| Google Plus + |  |  |  |
| YouTube |  |  |  |
| Snapchat |  |  |  |
| Other _________ |  |  |  |

1. Have you ever seen health messages promoted on any of the following social media platforms (tick all that apply):

Twitter
Facebook
Pinterest
Tumblr
Instagram
Linkedin
Google Plus +
YouTube
Snapchat
Other, please specify: _______________

1. Have you ever engaged (liked, commented, saved, shared) with health messages on social media?

Yes

No

1. How likely are you to trust health messages you read on social media?

Very unlikely
Unlikely
Neither likely nor unlikely
Likely
Very likely

**Thank you for completing this survey
Please follow the link below and enter your email address to be in with a chance of winning £20 in Love2shop vouchers.**

**Thank you for completing our survey**

**Please enter your email address below to be in with a chance of winning one of our £20 gift vouchers:**

**_______________________________________________________________**

Do you consent to being contacted in the future regarding participation in focus groups for this research project? *

Yes __

No __

*By consenting to being contacted, you are not automatically consenting to participate in the focus groups. The focus groups will last approx. 1 hour. A member of the research team will be in touch via email closer to the time detailing the procedure and providing full details regarding you and protection of data. Your participation is completely voluntary, and you will be free to withdraw at any time without giving a reason. Participants will be rewarded with a £10 gift voucher in exchange for participation in the focus groups.
